# Visualising the Cross-Level Relationships between Pathological and Physiological Processes and Gene Expression: Analyses of Haematological Diseases

**DOI:** 10.1371/journal.pone.0053544

**Published:** 2013-01-02

**Authors:** Masahiro Ono, Reiko J. Tanaka, Manabu Kano, Toshio Sugiman

**Affiliations:** 1 Immunobiology Unit, Institute of Child Health, University College London, London, United Kingdom; 2 Department of Bioengineering, Imperial College London, London, United Kingdom; 3 Department of Systems Science, Kyoto University, Kyoto, Japan; 4 Department of Human Coexistence, Graduate School of Human Environmental Studies, Kyoto University, Kyoto, Japan; UMDNJ-Robert Wood Johnson Medical School, United States of America

## Abstract

The understanding of pathological processes is based on the comparison between physiological and pathological conditions, and transcriptomic analysis has been extensively applied to various diseases for this purpose. However, the way in which the transcriptomic data of pathological cells relate to the transcriptomes of normal cellular counterparts has not been fully explored, and may provide new and unbiased insights into the mechanisms of these diseases. To achieve this, it is necessary to develop a method to simultaneously analyse components across different levels, namely genes, normal cells, and diseases. Here we propose a multidimensional method that visualises the cross-level relationships between these components at three different levels based on transcriptomic data of physiological and pathological processes, by adapting Canonical Correspondence Analysis, which was developed in ecology and sociology, to microarray data (*CCA on Microarray data, CCAM*). Using CCAM, we have analysed transcriptomes of haematological disorders and those of normal haematopoietic cell differentiation. First, by analysing leukaemia data, CCAM successfully visualised known relationships between leukaemia subtypes and cellular differentiation, and their characteristic genes, which confirmed the relevance of CCAM. Next, by analysing transcriptomes of myelodysplastic syndromes (MDS), we have shown that CCAM was effective in both generating and testing hypotheses. CCAM showed that among MDS patients, high-risk patients had transcriptomes that were more similar to those of both haematopoietic stem cells (HSC) and megakaryocyte-erythroid progenitors (MEP) than low-risk patients, and provided a prognostic model. Collectively, CCAM reveals hidden relationships between pathological and physiological processes and gene expression, providing meaningful clinical insights into haematological diseases, and these could not be revealed by other univariate and multivariate methods. Furthermore, CCAM was effective in identifying candidate genes that are correlated with cellular phenotypes of interest. We expect that CCAM will benefit a wide range of medical fields.

## Introduction

In order to fully understand pathological processes in clinical settings at the genomic level, it is necessary to compare the transcriptomes of pathological processes in individual patients with those of the physiological processes of normal counterparts. Although transcriptomic analysis has been extensively applied to various diseases, the way in which the transcriptomic data of pathological cells relate to the transcriptomes of normal cellular counterparts has not been fully explored, and may provide new and unbiased insights into the mechanisms of these diseases. To achieve this, it is necessary to develop a method to simultaneously analyse components across different levels, namely genes and physiological and pathological processes (e.g. normal and abnormal cellular phenotypes). It is anticipated, if successful, this approach will reveal hidden relationships between pathogenesis, developmental mechanisms, and gene regulation.

Gene signature has been a most commonly employed approach to address this type of problem. A number of methods have been proposed to measure the degree of inclination towards a certain signature in individual disease samples: correlation coefficient to the average gene expression of the signature genes [1], [2]; the median fold change [3]; the (weighted) sum of the expression levels of signature genes [4], [5]. Sandberg et al developed a method using Singular Value Decomposition (SVD) to measure the similarities between cancer subtypes and cell lines [6], providing univariate scores for individual cell lines. Although these approaches are easy to deal with and can be understood intuitively, there is a pitfall when they are applied to compare disease and normal cellular phenotypes: it cannot be assumed that the two cell signatures to be analysed are independent from each other. For example lymphoid and myeloid signatures cannot be equally compared and therefore the analysed results of these gene signature scores should not be plotted on the same plot, as the relationship between these two signatures is unknown. This fundamental problem complicates comparisons between multiple gene expression signatures of different haematopoietic cell populations. Considering that haematopoietic cells are classified into tens of different populations by cell lineage and developmental stage, and that each cell population is closely related to others [7], those existing methods are apparently insufficient for obtaining an integral view. Therefore, multidimensional analysis is required to effectively address this problem.

Among multidimensional analysis methods, principal component analysis (PCA) is most commonly used to analyse the relationships between samples, although PCA is vulnerable to the addition of subtle phenotypes and aberrant samples, and is more suitable for visualising data structure [8], [9]. In addition, although it may be more straightforward to estimate the identity of cells by directly comparing their transcriptomes with other microarray datasets using multivariate analysis, for example using Multidimensional Scaling [10], [11], this is often not successful because large between-experimental variations can easily dominate relatively small differences in gene modification between experimental and control groups even with meta-analysis methods. Considering that the variations between malignant cells and normal cells are generally much bigger than between-group variations of different normal cell phenotypes, as often seen in the analysis using hierarchical clustering [12], a new multidimensional approach is required to make a direct comparison of malignant cell phenotypes and their corresponding normal counterparts.

Thus, in order to reveal the cross-level relationships between diseases, genes, and normal cells, we have adapted a multidimensional approach, Canonical Correspondence Analysis (CCA), to microarray data. Currently, CCA is widely used in ecology and social science, as it can simultaneously analyse two totally different types of data – one as response data and another as explanatory data, revealing the relationships between these two data [9]. CCA is a variant of Correspondence Analysis (CA), which has previously been employed to analyse a single microarray dataset, visualising the associations between samples (arrays) and genes in single datasets [13], [14]. Baty *et* *al* reported a method using a variant of CCA for the analysis of microarray expression data with respect to binary response data [15]. As far as we know, the present study is the first to adapt CCA so as to simultaneously analyse two microarray data, which is designated as *CCA on Microarray data* (CCAM).

In order to examine the validity and efficiency of our method, we have analysed two haematological disorders: leukaemias and myelodysplastic syndromes (MDS). Table 1 is the summary of microarray datasets used in this study.

**Table 1:** Summary of microarray datasets used in this study.

ID	Study design	#samples	Microarray platform	Ref
GSE2779	Purified CD34^+^ progenitor cells from normal karyotype, low blast count MDS patients,age-matched controls and patients with non-MDS anaemia	28	HG-U133A	[49]
GSE13159	BM or blood samples of acute and chronic leukaemia patients	2096	HG-U133 plus 2	[29]
GSE15061	BM samples from MDS and non-leukaemia; AML data were not used in this study	233	HG-U133 plus 2	[22]
GSE24759	Flow-sorted 38 haematopoietic cell populations; Pooled samples from 4 to 7 independent donors	211	U133AAofAv2	[30]

Haematological disorders are classified and understood by referring to normal haematopoietic cell differentiation. Leukaemias are classified on the basis of the cell type involved and the state of maturity of the leukaemic cells, and categorized into major four groups: acute lymphoblastic leukaemia (ALL), acute myeloid leukaemia (AML), chronic lymphocytic leukaemia (CLL), and chronic myelogenous leukaemia (CML) [16]. The classification of leukaemias has been further developed by assigning leukaemic cells to normal haematopoietic cell counterparts based on morphology, cytochemistry, immunophenotype, genetics and clinical features, so as to define clinically significant disease entities [17], [18]. This framework is based on the well-known hypothesis that the genetic lesions of leukaemia result in a block of differentiation (maturation arrest) that allows leukaemic cells to continue to proliferate and/or prevents the terminal differentiation and apoptosis seen in normal white blood cells [19].

MDS are a group of clonal haematopoetic disorders marked by ineffective haematopoiesis, peripheral cytopenias, and an increased risk of transformation to AML [20]. MDS have been classified into subgroups, and individual patients are scored, in order to predict prognosis, especially for assessing the risk of leukaemic transformation. The International Prognostic Score System (IPSS) for MDS is composed of three factors: blasts in bone marrow (BM), karyotype, and cytopenia, and higher scores are associated with poorer prognosis [21]. The World Health Organization (WHO) classification of MDS is based on morphologic evaluation of bone marrow cells and genetic abnormalities, and classifies MDS into 6 major subtypes: refractory anaemia (RA, or Refractory cytopenia with unilineage dysplasia (RCUD)), refractory anaemia with ring sideroblasts (RARS), and refractory cytopenia with multi-lineage dysplasia (RCMD), and 5q-syndrome (MDS associated with isolated del(5q)), and refractory anaemia with excess blasts (RAEB-1 [blasts 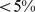] and RAEB-2 [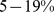 blasts]) [22], [23]. Blast percentage of more than 20% is defined as AML, and reasonably, RCMD and RA show better prognosis with longer leukaemia-free survival than RAEB-1 and RAEB-2 [24], [25].

Genome-wide gene expression analysis (transcriptomic analysis) has been extensively used for improved understanding of the diagnosis, prognosis, and pathogenesis of these haematological diseases [22], [26]. In these transcriptomic studies, gene expression signature (or, gene expression profiles [GEP]) has been most commonly used to classify haematologic diseases and predict prognosis [26]. Gene expression signature is typically composed of tens to hundreds of genes, so that all these genes stably contribute to classify samples in cross-institutional settings [4], [27]. Hierarchical clustering is most often employed in analyses using gene expression signatures to classify samples into disease subtypes [28].

## Results

### Analysis 1: Leukaemia

Based on the assumption that leukaemia is classified by referring to normal haematopoietic cell differentiation, we attempted to analyse using transcriptomic data the relationships between leukaemia disease samples and normal haematopoietic lineage cells. We aimed in this analysis to determine the transcriptomic identities of individual leukaemia patients by analysing a transcriptomic dataset of leukaemia (GSE13159 [29]) and that of haematopoietic cell differentiation (GSE24759 [30]). As this is the first exemplary analysis using a univariate approach by gene signature, we also show why we need to introduce a multidimensional method. In the subsequent section, we demonstrate how CCAM is applied to microarray datasets, and examine the validity of the method by addressing haematologically well-known relationships between pathological and physiological processes.

#### A univariate approach using gene expression signature

First, we employed a univariate approach to address this problem, using provisional gene signatures of haematopoietic cell populations (see Methods). Here, we aim to score individual disease samples by the degree of maturation into each cell population. Given that some haematopoietic cell populations may be too similar to each other to provide meaningful results that discriminate disease samples, hierarchical clustering was used to cluster haematopoietic cell gene signatures based on their correlations to individual disease samples (Fig. S1). Based on this clustering, we chose four distinct (classified in different groups) gene signatures from relatively immature cells (proxy to haematopoietic stem cells [HSC]): granulocyte-monocyte progenitor (GMP; CD34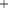 CD34
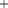
CD38
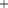
CD45RA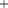), neutrophilic metamyelocyte (NM; CD34
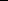
SSC
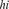
CD45
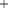
CD11b
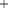
CD16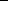), immature B cells (Pro-B; CD34
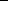
CD10
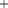
CD19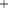) and mature B cells (Mat-B; mature B cells with class switched; CD19
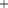
IgD
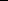
CD27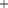) [30]. We analysed the distribution of gene signature scores of disease samples for these cell populations. As shown in Fig. S2, CLL showed high correlations with the signature of Mat-B (Fig. S2a), while CML and AML showed higher correlations with those of NM and GMP (Fig. S2b and S2c). Generally, ALL showed higher correlations with the signature of Pro-B (Fig. S2d). These results seemed haematologically appropriate, considering the immunophenotype of these leukaemia subtypes [31]–[34]. It was, however, unclear how these four results in Fig. S2 were related.

#### Cross-level relationships between leukaemia subtypes, haematopoietic cell differentiation, and genes by CCAM

**Figure 1:**
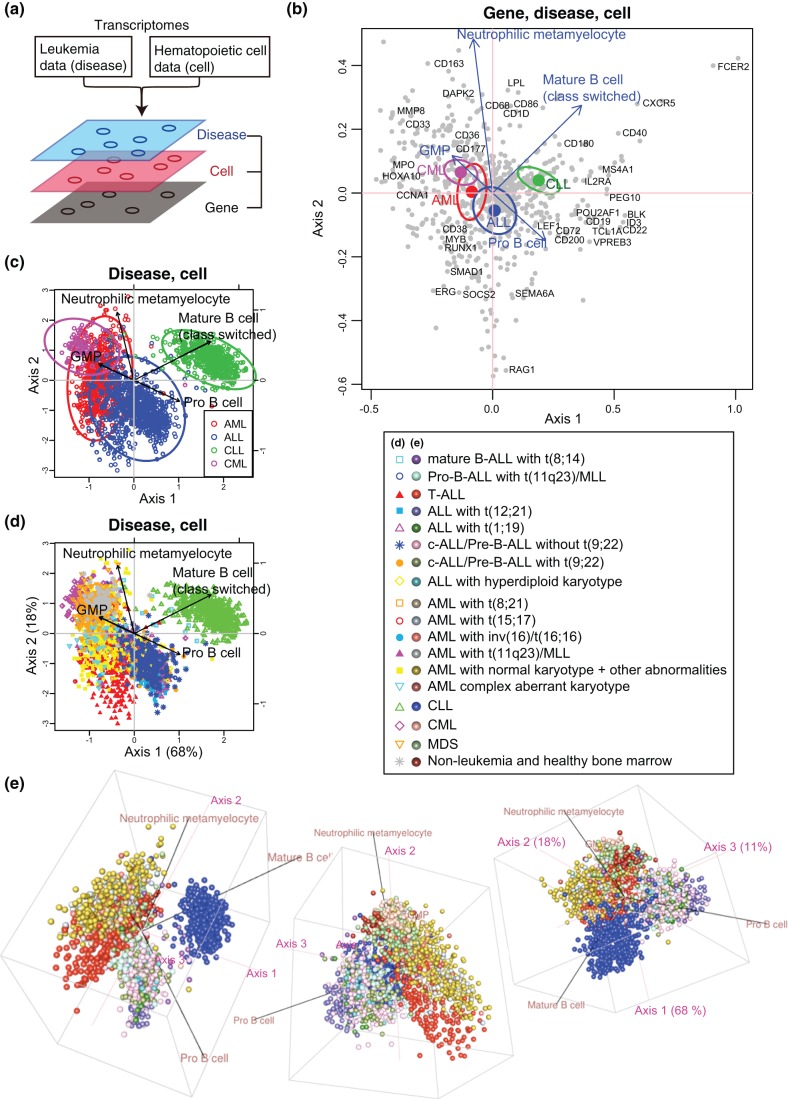
CCAM of transcriptomic data of leukaemias with haematopoietic cell differentiation as explanatory variables. Leukaemia data were analysed with those of haematopoietic cell populations at distinct differentiation states (Granulocyte-monocyte progenitor [GMP], Neutrophilic metamyelocyte, Pro-B cell, and Mature B cell class switched,). (a) Schematic presentation of CCAM. Transcriptomic datasets of leukaemias (including AML, CML, ALL, and CLL) and haematopoietic cells were processed by CCA and the cross-level relationships between components at three different levels, namely disease, cell, and gene, were analysed. (b) All three levels are shown on a map (CCA triplot). Centroids of disease samples are shown by large closed circles, and 95% confident intervals (CI) are indicated by ellipsoids. Genes are shown by closed grey circles, and well-known genes that are key for either leukaemia or haematopoietic cell differentiation are annotated. Haematopoietic cells are represented by blue arrows, towards which genes and diseases that are closely related to the corresponding cell are aggregated. (c, d) The levels of disease and cell are shown. (c) Individual disease samples are shown in addition to 95% CI. (d) Two-dimensional plot of disease samples and haematopoietic cell populations. The amount of information (eigenvalue) retained in each axis is 68% and 18% of the total variation (precisely, *constrained inertia*, see Methods) for Axis 1 and 2, respectively. (e) Three-dimensional plots of disease samples and haematopoietic cell populations. The amount of information (eigenvalue) retained in each axis is 68%, 18%, and 11% (of the constrained inertia) for Axis 1, 2, and 3, respectively. See legend for symbols and colours in (d) and (e).

The results above indicated that it was necessary to simultaneously analyse components at three different levels: genes, normal haematopoietic cells, and individual disease samples. To achieve this, we have developed a new multidimensional and canonical analysis of two microarray datasets by adapting Canonical Correspondence Analysis, which was developed in ecology and sociology, to microarray analysis (we designate the method as *CCA on Microarray data, CCAM*) (see Methods, Fig. 1a). Briefly, in our application of CCAM, pathological data (disease data) are treated as response data, and physiological data (normal haematopoietic cell differentiation) are used as explanatory variables (environmental variables), and thereby we aim to reveal the relationships between gene expression and pathological and physiological processes. Assuming that leukaemias are classified by referring to normal haematopoietic cell differentiation, CCAM is expected to assign individual disease samples to most correlated normal counterparts. We used the four representative haematopoietic lineage cells that were analysed in the gene signature approach in Fig. S2 (GMP, NM, Pro-B, and Mat-B).

We have employed a map approach in our method in order to avoid the pitfalls of simultaneously analysing the complex relationships between components at three different levels [9]. CCAM provides a map that shows the correlations between genes, normal haematopoietic cells, and disease samples. In other words, the more correlated, the nearer components are positioned on the map [9]. Fig. 1b shows all the components that were analysed at all the levels (gene, normal cell, and disease). On the map, CLL showed high correlations with Mat-B, and not with Pro-B and myeloid cells, compatible with the fact that the phenotype of CLL is similar to antigen-experienced B cells rather than immature B cells [31], [34] (Fig. 1b and 1c). Although the number of samples is small, mature B cell-ALL (mature B-ALL) showed a clear correlation with CLL and Mat-B [33] (Fig. 1d), which is also a reasonable result. On the other hand, ALL (excluding T cell-ALL [T-ALL] and mature B-ALL) showed higher correlations with the signature of Pro-B, which is consistent with the immunophenotype of non T cell, immature B cell ALL [32], [33]. CML and AML showed higher correlations with the signatures of NM and GMP, and comparing with AML, CML was more distinct from lymphocytic leukaemias (ALL and CLL) and deviated more to the direction to which NM and GMP were correlated (Fig. 1b–1e), confirming a more differentiated granulocytic phenotype of CML than AML. T-ALL was distinct from other ALL, and positioned between B-ALL and AML (Fig. 1d–1e).

By analysing CCA triplot at the gene level, B cell- and B-leukaemia-related genes have high (positive) scores in axis 1, while genes related to myeloid cell differentiation and myeloid leukaemia have low (negative) scores (Fig. 1b). Myeloid genes such as *MMP8* and *CD33* are in quadrant I (Axis1
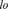
Axis2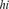), which is correlated with myeloid lineage NM and GMP. Genes related to nave or immature B cell (e.g. *POU2AF1, CD19, ID3, VPREB3, RAG1*) are apparently enriched in quadrant III (Axis1
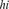
Axis2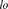), which is correlated with Pro-B. Genes related to mature, antigen-experienced B cells (e.g. *CD40, CD86*) are found in quadrant II (Axis1
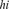
Axis2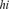), which is correlated with Mat-B. The associations of components at the three different levels could be observed in this analysis. For example, in quadrant II, CCL and mature B cells are correlated with *FCER2* (*CD23*, FcR*ε*II), *CD180* (*RP105*), and *CXCR5* (Fig. 1b). In fact, increased expression of these genes is characteristic in CLL and also associated with maturation of B cells [35]–[39]. Interestingly, quadrant IV (Axis1
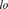
Axis2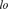), which is not annotated by haematopoietic cells but correlated with AML and T-ALL, includes *RUNX1*, *ERG*, and *MYB*, which have well-established roles in AML and early haematopoietic differentiation including myeloid and T-lymphocyte lineages [40]–[43] (Fig. 1b).

Thus, the map analysis in Fig. 1 can be summarised as follows: Axis 1 represents“myeloid cells vs. B lymphocyte”, while Axis 2 represents “immature vs. mature cells”. Individual leukaemia samples and gene expression were successfully characterised on this map. The analysis of variation (precisely, *inertia* [9]; see Methods) showed that Axis 1, 2, and 3 comprised 68%, 17%, 11% of variations, respectively. This means that the leukaemia data that was interpretable by the haematopoietic cell data was mostly visualised (85% and 97% of the information in the constrained data in Fig. 1d and 1e, respectively), and that the difference between myeloid and lymphocytic lineages dominated that of the maturity of cells in this dataset.

Next, we further analysed the phenotypes of AML subtypes by CCAM (Fig. S3). We included in the analysis the cell populations of the myeloid lineage that are relevant in AML, namely, Common myeloid progenitor (CMP), Colony forming unit-monocyte (CFU-M), Neutrophilic metamyelocyte, and mature Neutrophils. CCAM classified AML subtypes with the features of the granulocytic and monocytic lineages (Fig. S3). CCAM showed that the subtypes AML with 11q23/*MLL* and AML with inv(16)/t(16;16) were more associated with CFU-Monocyte than other granulocyte lineage cells, which is consistent with the facts that these subtypes are morphologically more correlated with the monocytic lineage [44], [45]: a study showed that a majority (81%) of AML with 11q23/*MLL* showed an involvement of the monocytic lineage [44]; AML with inv(16)/t(16;16) has the fusion gene *CBF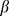/MYH11*, and is morphologically associated with the French-American-British (FAB) AML-M4 subtype (acute myelomonocytic leukaemia with an abnormal eosinophil component)[45]. In addition, CCAM showed that the subtypes AML with t(15;17) and AML with t(8;21) were more related to Neutrophil, which is consistent with their morphological associations with the granulocytic lineage: AML with t(15;17) has the *PML-RARA* fusion gene and corresponds to the FAB M3 subtype (acute promyeolocytic leukaemia) 46,47; AML with t(8;21) has the fusion gene *AML1(RUNX1)/ETO* and corresponds to the AML-M2 (acute myeloid leukaemia with maturation) [48]. See File S1 and Fig. S4 for the further analysis of these two datasets.

### Analysis 2: Myelodysplastic Syndromes (MDS)

The analyses above showed that CCAM successfully revealed known relationships between leukaemia, haematopoietic cell differentiation, and genes in a concise and transparent way. In this section, we examined whether the approach was effective in generating and testing hypotheses, and questioned whether this method could provide meaningful insights into clinical problems. Accordingly, we analysed two independent MDS datasets. The first analysis was carried out in order to generate a new hypothesis. We then tested the hypothesis by analysis of another independent dataset.

#### Analysis for hypothesis-generation: comparison of MDS and normal bone marrow (BM)

We analysed the transcriptomic data by Sternberg et al (GSE2779 [49]) along with that of haematopoietic cell differentiation (GSE24759). Sternberg et al showed that CD34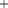 progenitor cells from normal-karyotype, low-blast-count MDS patients consistently showed decreased expression of B-cell lineage-affiliated genes [49]. We attempted to confirm this result, while obtaining a bigger picture using not only the data of immature B cells (Pro-B) but also those of other progenitors including MEP (megakaryocyte-erythroid progenitor), GMP, and CMP, and haematopoietic stem cells (HSC). In order to find the unique features of MDS BM using the relatively small number of disease samples analysed in this dataset, we filtered genes using the disease data.

**Figure 2:**
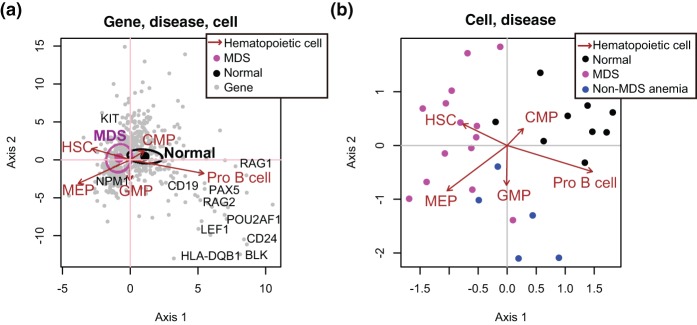
CCAM of MDS transcriptomic data of CD34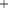 cells from MDS, non-MDS anaemia, and normal BM, analysed together with those of haematopoietic cell differentiation. Microarray data of CD34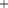 cells from MDS, non-MDS anaemia, and healthy controls were analysed by CCAM using five haematopoietic cell populations (Haematopoitic stem cell [HSC], Megakaryocyte-erythroid progenitors [MEP], Common myeloid progenitor [CMP], Granulocyte-monocyte progenitor [GMP], Pro-B cell) as explanatory variables. Genes were filtered by MDS data using an empirical Bayes t-statistic [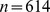]). (a) CCA triplot. Centroids of MDS and normal CD34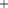 cells are shown by large closed circles, and 95% confident intervals (CI) are indicated by ellipsoids. Genes are shown by closed grey circles, and well-known genes that are key for either MDS or corresponding haematopoietic cells are annotated. Axis 1 indicates the direction to which the variation (inertia) is the largest (87% of the total variation [constrained inertia]). Axis 2 has the second largest inertia (7%). (b) Individual disease samples are shown without genes to clearly show the relationships between disease samples and haematopoietic cell populations.

CCA triplot showed that axis 1, which was composed of the largest variation in the dataset, is primarily represented by the difference between MDS and normal BM as well as that between HSC/MEP and CMP/Pro-B. As Sternberg et al reported, MDS samples had negative correlations with Pro-B (Fig. 2a). In addition, CCA triplot showed that MDS samples had positive correlations with HSC and MEP. Although the number of samples is small, non-MDS anaemia samples were in the middle of MDS and normal BM in axis1 (Fig. 2b). Interestingly, *KIT* and *NPM1*, the mutations in which are suggested to play roles in leukaemic transformation [50], [51], were correlated with MDS and HSC/MEP. B cell-related genes including *POU2AF1*, *PAX5*, and *CD19*, were associated with normal BM as reported [49] (Fig. 2a).

#### Examination of hypothesis: BMs from high-risk MDS patients showed the deviation towards HSC/MEP at transcriptomic level

Based on these findings, we generated a hypothesis that MDS patients with higher correlations with both HSC and MEP (and negative correlation with Pro-B) had a higher risk for leukaemic transformation. To test this hypothesis, we have applied CCAM to another independent dataset of MDS that is composed of only MDS patients (without normal), and analysed the results in conjunction with the clinical data (GSE15061 [22]).

**Figure 3:**
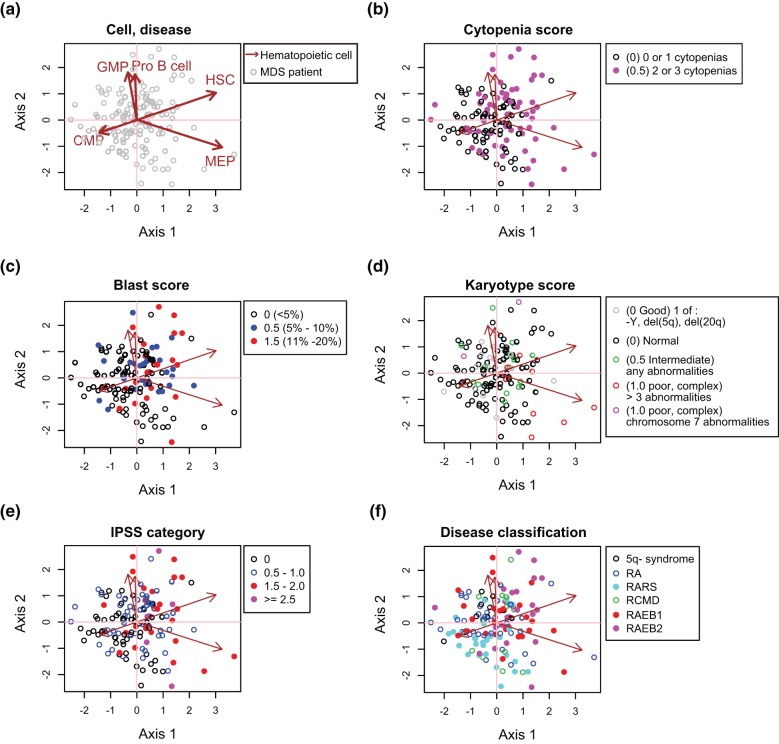
Hypothesis-testing by CCAM using another independent MDS dataset. Microarray data of MDS patients (without normal BM) were analysed using five haematopoietic cell population (HSC, MEP, CMP, GMP, Pro-B cell) as explanatory variables. Genes were filtered only by haematopoietic cell data (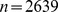), therefore this is an unsupervised analysis in terms of MDS disease data. (a) CCAM result showing the disease and cell levels. Axis 1 indicates the direction to which the variation (inertia) is the largest (55% of the total variation [constrained inertia]). Axis 2 has the second largest inertia (21%). MDS patient samples are positioned according to the correlations with five haematopoietic cell populations in terms of gene expression. (b–d) The following clinical data of individual disease samples were superimposed on the map in (a): (b) cytopenia score; (c) blast score; (d) karyotype score; (e) IPSS category; and (f) disease classification.

First, using CCAM, we determined the relationships between individual MDS patients and haematopoietic cells based on their transcriptomes (Fig. 3a). Next, we superimposed clinical data onto this plot. Interestingly, MDS patients with high scores in IPSS scores (cytopenia, and blast) had higher values in axis1 compared with those with low scores (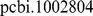 for cytopenia [cytopenia score 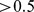 vs. 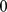], 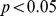 or 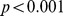 for blast [blast 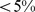 vs. blast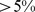 or 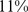, respectively], Fig. 3b). IPSS category itself had a similar tendency: individuals with IPSS 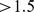 had significantly higher scores in axis 1 (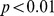). According to the WHO classification, RAEB-2 had significantly higher axis 1 scores than others (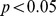). Importantly, principal component analysis (PCA) could not reveal these characteristics of MDS patients (Fig. S5), demonstrating the value of CCAM.

#### CCAM created a new scoring system that has a prognostic value and biological relevance in haematopoietic cell development

**Figure 4:**
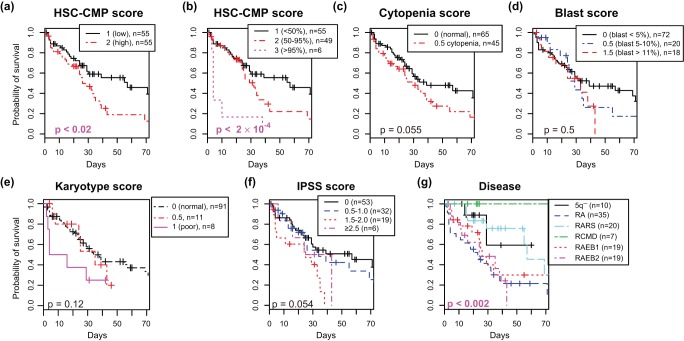
Kaplan-Meier curves for overall survival for MDS patients according to the HSC-CMP score(a, b), which was made by CCAM, and the well-established classifications (c–g). Patients were stratified into 2 to 6 groups by the followings: (a) HSC-CMP score, two groups (1: 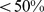 and 2: 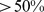). (b) HSC-CMP score, three groups (1: 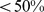, 2: 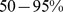, and 3: 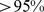). (c) Cytopenia score. (d) Blast score. (e) Karyotype score. (f) IPSS score. (g) Disease classification. P values are by log-rank test.

**Figure 5:**
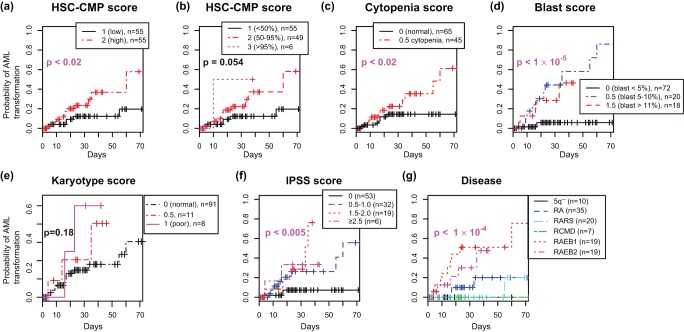
Kaplan-Meier curves for time to AML transformation for MDS patients according to the HSC-CMP score(a, b), which was made by CCAM, and the well-established classifications (c–g). Patients were stratified into 2 to 6 groups by the followings: (a) HSC-CMP score, two groups (1: 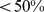 and 2: 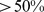). (b) HSC-CMP score, three groups (1: 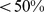, 2: 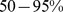, and 3: 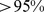). (c) Cytopenia score. (d) Blast score. (e) Karyotype score. (f) IPSS score. (g) Disease classification. P values are by log-rank test.

The results in Fig. 3 suggest that a positive association with HSC/MEP and a negative association with CMP has a prognostic value. Thus, using top ranked genes (top 100 and bottom 100 genes by the *wa* score of CCAM, see Method), we analysed the relationships between individual patients and their associations with the four haematopoietic cell populations, and thereby established a scoring system for MDS patients (designated as *the HSC-CMP score*). As expected, genes with high HSC-CMP scores were specific to HSC/MEP, while those with low scores were specific to CMP/GMP (Fig. S6). MDS patients were stratified into two or three groups by the HSC-CMP score, and two to four groups by well-established prognostic scores and the disease categories in the WHO classification. Kaplan-Meier survival analysis showed that the HSC-CMP score had prognostic values for overall survival: patients with scores above the 50th percentile showed worse survival (Fig. 4a 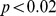) and those above the 95th percentile had the worst prognosis by log-rank test (Fig. 4b 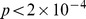). While the IPSS and cytopenia scores showed p-values just above the significant level(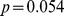 and 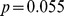, respectively), disease categories showed significant difference between patient groups (Fig. 4g, 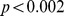). Similarly, Kaplan-Meier survival analysis for time to AML transformation showed that patients who scored above the 50th percentile had worse prognosis (Fig. 5a, 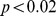), while the stratification of patients into three groups by the HSC-CMP score was less significant (Fig. 5b, 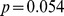). Regarding the AML transformation, reasonably, blast score and disease categories showed the lowest p-values (Fig. 5d and 5g, 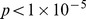 and 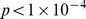, respectively).

To further address the significance of the HSC-CMP score, we employed a Cox proportional hazard regression analysis for overall survival and time to AML transformation. The univariate Cox analysis showed that the HSC-CMP score and the IPSS score were significant both for time to AML transformation (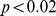 and 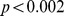, respectively) and for overall survival (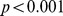 and 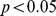, respectively, Table 2). Cytopenia and Blast scores were also significant for time to AML transformation (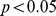 and 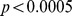, respectively). Next, we performed a multivariate Cox regression analysis with the IPSS score and the HSC-CMP score (three stratified groups), which showed that the HSC-CMP score remained significant for overall survival but the IPSS score did not (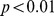, hazard ratio [HR]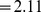; and 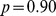, HR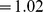; respectively), while the IPSS score remained significant for time to AML transformation but the HSC-CMP score did not (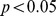, hazard ratio [HR]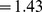; and 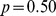, HR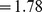; respectively). Lastly, we performed a multivariate Cox regression analysis with the HSC-CMP score, Cytopenia score, Blast score, and Karyotype score. The analysis for time to AML transformation did not show any statistically significant results except that Blast score was significant (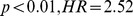). The analysis for overall survival showed that the HSC-CMP score had the largest impact (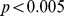, HR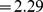), and other scores failed to show significance (Table 2). These results suggest that although the HSC-CMP score was not independent from the IPSS and other scores, it was a dominant prognostic factor for overall survival.

**Table 2:** Results of the univariate and multivariate Cox proportional hazard regression analysis.

	Time to AML transformation					Overall survival				
Covariate	P	Hazard ratio		Confidence interval		P		Hazard ratio		Confidence interval
**Univariate hazard ratios**										
HSC-CMP score	<.02^*^		2.57^*^		1.17–5.64		<.001^*^		2.15^*^	1.37–3.38
IPSS score	<.002^*^		1.99		1.29–3.06		.03^*^		1.34	1.03–1.74
Cytopenia score	<.05^*^		2.92^*^		1.15–7.41		.059		1.66	0.98–2.80
Blast score	<.0005^*^		2.85^*^		1.64–4.97		.27		1.21	0.86–1.70
Karyotype score	.08		1.84		0.93–3.62		.061		1.49	1.00–2.26
**Multivariate hazard ratios**										
HSC-CMP score	.50		1.43		0.51–4.03		<.01^*^		2.11^*^	1.20–3.71
IPSS score	<.05^*^		1.78		1.03–3.07		.90		1.02	0.72–1.45
**Multivariate hazard ratios**										
HSC-CMP score	.86		1.10		0.37–3.26		<.005^*^		2.30^*^	1.29–4.10
Cytopenia score	.89		0.91		0.24–3.40		.84		1.08	0.54–2.16
Blast score	.01^*^		2.82^*^		1.27–6.27		.28		0.79	0.51–1.22
Karyotype score	.92		1.04		0.50–2.18		.33		1.25	0.79–1.98

HSC-CMP score is the score made by CCAM, and stratified patients into three groups (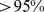, 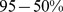, and 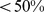). Cytopenia, Blast, and Karyotype scores are the scores that constitute the IPSS score. These scores were examined for their impacts on overall survival and time to AML transformation.

P
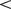
0.05 or hazard ratio 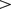
2.0.

## Discussion

CCAM has provided novel insights into the cross-level relationships between gene expression and pathological and physiological processes, which could not be obtained by the analysis at each single level. Importantly, many medical problems require the analysis of disease samples in the context of some particular biological processes (e.g. cell differentiation), which are most often multidimensional in nature and are often not straightforward. CCAM has effectively solved this type of problems by analysing two independent transcriptomic data. Visualisation of the analysed results has made it transparent which cell populations are being compared for the relationship with disease samples. In addition, CCAM allows the exploration of novel molecular mechanisms that are highly associated with particular cell and/or disease. For example, in Fig. 1, CCAM has identified known genes that had roles in haematopoietic cell differentiation and leukaemia. This result suggested that other genes that are associated with (in juxtaposition to) these known genes and with particular diseases and cells are reasonably good candidates for undefined molecular mechanisms of these diseases and cells, considering the nature of the underlying algorithm, Correspondence Analysis [9], [14]. Thus, CCAM with the map approach is useful for generating hypotheses with least assumptions, and is expected to lead to hypothesis-driven studies. In addition, we used CCAM effectively to test a hypothesis on the transcriptomic characteristics of MDS patients. Furthermore, the proposed approach can identify individuals with worse clinical outcomes, and infer the mechanisms underlying poor prognosis, as shown by the analysis of MDS. The clinical utility of this approach is thus demonstrated.

### New biological insights from CCAM: the positive correlation of transcriptomes of high risk MDS patients and those of HSC/MEP (and the negative correlation of MDS with CMP)

Consistent with a previous report [49], MDS samples showed decreased or negative correlations with the process of early B cell differentiation compared with healthy controls. In adddition, our analysis has revealed that MDS samples are more correlated with the processes of both HSC maintenance and MEP differentiation. Interestingly, while the process of early B cell differentiation did not show correlation with the severe phenotype of MDS, the transcriptomic statuses of HSC and MEP showed remarkable correlations with it (Fig. 3). The leukaemic transition of MDS is widely known to be associated with the immaturity (the acquisition of stem-ness [HSC]) [22], [23], while the association between MDS and MEP (in comparison with myeloid/lymphoid differentiation) has not been recognised. This is the power of the new approach: to simultaneously and fairly consider multiple phenotypes, providing an integral view on the system.

As RNA for hybridization was extracted from unsorted, mononuclear BM cells from MDS patients in this dataset (GSE15061) [22], the result should be interpreted considering possible compensatory mechanisms in BM by non-MDS cells. It is noticed, however, that another dataset, GSE2779, analysed purified CD34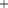 progenitor cells, resulting in a similar conclusion. In addition, our results showed that RARS BM, which characteristically show hyperplastic ineffective erythropoiesis [52], did not have positive correlations with MEP and HSC, and that 5q-syndrome, which typically shows normal to increased megakaryocytes [23], did not show positive correlations with MEP. Rather, 5q-syndrome and RARS showed lower values in Axis 1, and were more correlated with CMP (Fig. 3f). Given that the largest variation can be observed in axis 1 of CCAM results, these results indicate that the distinct feature of BM from severe MDS patients dominated the variations in MDS including those compensatory mechanism and secondary responses. Interestingly, a recent report showed that decreased expression of erythroid-specific genes was correlated with the responsiveness to the thalidomide derivative lenalidomide in patients with 5q-syndrome, which is the most homogenous subtype of MDS [53]. Our results, along with the results of this study, suggest that MDS with higher correlations with the erythroid lineage (and HSC) are more difficult to treat with lenalidomide.

Importantly, the HSC-CMP score showed a prognostic value for overall survival (Fig. 4–5 and Table 2). Given that the HSC-CMP score was correlated with the characteristic gene expression in HSC/MEP and CMP/GMP (Table S1 and Fig. S6), those genes can be investigated to elucidate the molecular mechanisms of MDS pathogenesis in relation to the cellular and differentiation processes of these haematopoietic cell populations. These findings above suggests that disease progression in MDS is accelerated by the aberrant utilisation of the molecular mechanisms of erythroid/megakaryocyte (MEP) differentiation and stemness (HSC) and the loss of the mechanisms of CMP and/or GMP. The investigation of these processes may provide clues to identify new therapeutic targets that improve overall survival. Thus, CCAM provides biologically effective and meaningful solutions because it analyses simultaneously and cohesively two different phenotypic levels -in this case, MDS pathology and normal haematopoietic cell development.

Our analyses using transcriptomic data obtained from batch samples have revealed the transcriptomic identities of the phenotype of dominant cells or ‘average’ cells in the context of normal haematopoietic cell differentiation. It is known that variations can occur within individual cancers, in which the cancer cells often have a range of functional properties and diverse expression of markers [54]. In addition, it is thought that leukaemia has a hierarchical organization similar to that of normal haematopoiesis in which there is a rare subpopulation with limitless self-renewal potential (leukaemic stem cells) that gives rise to progeny that lack such potential [55]. Considering this, in conjunction with the use of our method, gene expression analysis at the single cell level will be the key to reveal further relationships between normal cells, cells of origin, and leukaemia stem cells. CCAM provides the framework to analyse this type of data.

### Technical considerations on the use of CCAM

CCAM does not produce ready-to-go results, but provides a platform where the existing hypotheses are examined and new hypotheses are formed and generated. Depending on how explanatory variables are set and used, CCAM can be used for exploratory purposes in a data-oriented way (c.f. Fig. 1) or for examining the original hypothesis (c.f. Fig. 3). The map approach enables the comparisons of more than two variables, while the regression process in CCAM allows the analysis across two different experiments. These are advantages of CCAM but can mislead the analysis if inappropriately used. The users should be aware of the following two points. First, because the underlying algorythm, CCA, uses multiple regression, one needs to avoid the pitfalls of multiple regression when choosing explanatory variables: the number of explanatory variables should be less that that of samples, in order not to overfit the data to explanatory variables; and the interpretability of the results is directly dependent on the choice and quality of the explanatory variables [56]. Second, when the final result of CCAM has only a very small part of the original “information” (*i.e*. %Explained is very low [e.g. 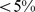], see Methods), interpretation should be cautious. Although the absolute value of %Explained does not reflect the biological relevance, if %Explained is comparable between analyses (see Methods), the results with larger %Explained values may be biologically more reasonable and straightforward.

**Figure 6:**
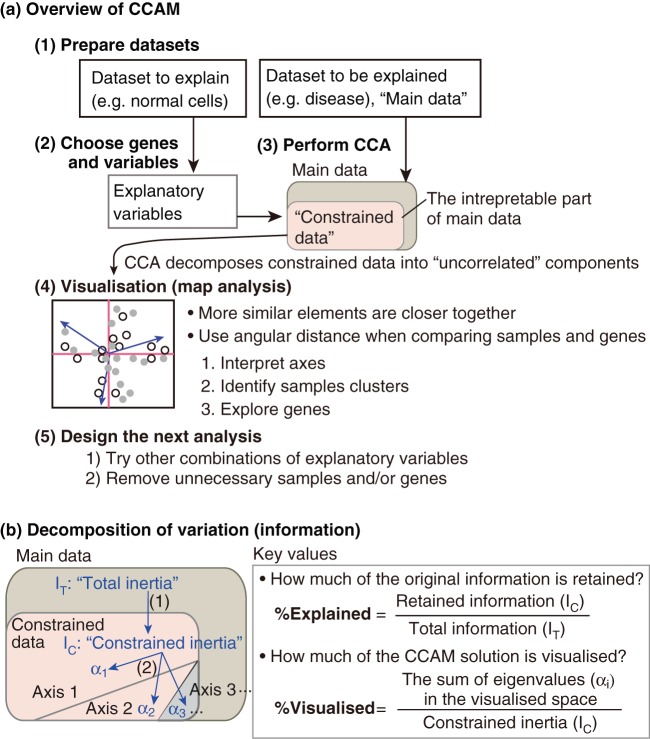
Schematic representation of CCAM and instructions for its practical usage. (a) Overview of CCAM. See Methods for the full instructions. (b) Schematic representation of the decomposition of variation (*inertia*). (1) Total inertia is divided into constrained and unconstrained inertias by regression of main data on explanatory variables. (2) Constrained inertia is distributed to different axes by singular value decomposition.

Importantly, the effective use of CCAM requires deep knowledge of both biology/medical science and multidimensional analysis. Because CCAM can help the process of hypothesis generation and testing, this method is best performed when biologists/medical scientists actively participate in the analysis. Readers with biological backgrounds are encouraged to understand the procedures of CCAM in Fig. 6, and those with bioinformatics/statistical knowledge can refer Fig. S7 for the theoretical background of CCAM, and use an R script of CCAM in File S1. If successfully applied, CCAM will allow one to address more complex questions which could not be done by conventional methodologies. The use of CCAM and related multidimensional methods will extend the power of experimental technologies, as it has benefited the field of ecology [56], [57]. Table 3 summarises the features of CCAM in comparison with univariate approaches including gene signature and multivariate/multidimensional approaches including PCA and CA.

**Table 3:** The features of CCAM and other univariate and multivariate/multidimensional methods for microarray analysis.

Method	Analysis for a single variable	Simultaneous analysis of ≥2 variables	Analysis across 2 different experiments	Exploratory analysis	Hypothesis driven analysis
Univariate^1)^	**√**		√		√
PCA		√		√	
CA		√		√	
CCAM (CCA)	**√**	√	√	√	√

Although the methods can be used for the application without “ticks” in some limited situations, the maximal productivity may be obtained by those with “ticks”.

1) Includes various methods for the signature approach. Commonly analysed using clustering methods.

Notably, CCAM has clarified in the analysis exactly what is compared and analysed, with the power of the map approach. Although it might be obvious to haematologists that HSC is correlated with the progression of MDS, CCAM revealed this without prior knowledge, and more importantly, showed that this statement was true only when patient samples were compared with not only HSC but also some more differentiated cells. CCAM identified that CMP was an appropriate cell population to be compared with HSC (c.f. Fig. 3). In addition, CCAM showed that MEP was correlated with MDS patients with worse prognosis at an equivalent level to HSC (Fig. 2–3). These led to the establishment of the HSC-CMP score, which had a prognostic value and biological relevance in haematopoietic cell development (Fig. 4–5, Table 2, Table S1). Such a complicated comparison of multiple phenotypes is important for a deep understanding of the system, but this is generally difficult by conventional approaches. The power of CCAM lies in its ability to deal with multiple phenotypic data based on quantitative measurements (*i.e*. gene expression).

## Conclusions

CCAM provides a practical solution for comparing multiple groups of samples with multiple cellular phenotypes. In addition, CCAM reveals hidden relationships between pathological and physiological processes and gene expression, providing novel clinical insights into haematological diseases. In fact, CCAM provided new insights such as the correlation of the severe phenotype of MDS and MEP, in addition to the known correlation with HSC. Furthermore, CCAM can be effectively used for exploring the genes that are correlated with cellular phenotypes of interest using the map approach (c.f. Fig. 1).

The strength of CCAM is its ability to explore datasets for hypotheses-generation using the map approach. Once a hypothesis is generated, we can test the hypothesis by this method using other datasets (c.f. Fig. 3) or by other methods such as statistical comparison of a few selected groups. The map-based approach is popular in social science and ecology [9], because in these research fields it is mandatory to analyse the relationships between many different, but closely associated qualitative data (e.g. different human attitudes [9], [58], species, and the analysis without visualisation can be misleading [9], [59]). In fact, qualitative data (e.g. disease classification, cell subtype) are becoming more and more complex in modern medicine with the emergence of genomics, and we believe that this type of approach is essential and should be incorporated into medical genomics. In addition, considering the generality of the method, the proposed approach can be applied to common problems in broad fields of molecular biology.

## Materials and Methods

### Canonical Correspondence Analysis on Microarray data (CCAM)

#### Conceptual definition of CCAM

CCA is a variant of Correspondence Analysis (CA), which is a key method in sociology, and is developed in ecology for identifying the relationships between ecological data at three different levels [9], [59]. We have chosen CCA because it can analyse the rows and columns of two transcriptomic data that are derived from two independent experiments with totally different experimental designs and materials. When these data are analysed by conventional approaches, the distance between different experiments (between-experiment variations) dominates important biological effects in the datasets, and compromises the direct comparison of leukaemic cells and normal haematopoietic cells. On the other hand, CCA looks at the intersection of the two completely different datasets (but obtained for the same genes) and thus avoids this problem. CCA in our method analyses the relationships between genes and disease samples in the context of haematopoietic cell differentiation (corresponding to *geological locations*, *species*, and *environmental variables*, respectively, by ter Braak [59]; gene expression levels correspond to frequencies). Note that in CCAM, CCA is applied to a matrix of data with genes in rows (observations, equivalent to *geological locations* by ter Braak) and cellular phenotypes in columns (variables, equivalent to *species* and *environmental variables* by ter Braak).

Briefly, first, CCAM projects the dataset of disease samples onto the data of haematopoietic cell differentiation, which are averaged for each cell population using scaled data with an average of zero and standard deviation of one for each gene (Fig. S7). Importantly, the gene expression data of haematopoietic cells represent the *environmental variables* that define the phenotypes of each cell population. Thus, CCAM analyses the interpretable part of the original disease dataset by haematopoietic cell data [9], [59]. Mostly the interpretable part (*%Explained*, see below) was 10–20% of the original data in the presented analyses [9]. Next, CCAM finds new axes by assigning numerical values to samples and genes so that the dispersion of samples is maximized [59].

#### Instructions on the practical usage of CCAM

Fig. 6a depicts how to use CCAM. (1) Prepare datasets. “Dataset to be explained” will be the data that analysed more ambiguous materials (e.g. disease samples) and are of most interest in the analysis. “Dataset to explain” will provide explanatory (environmental) variables, and be the one that analysed well-characterised materials (e.g. normal cells). (2) Choose genes using “dataset to explain” by some statistical method (e.g. a moderated t-test). Besides, prepare explanatory variables from “Dataset to explain” by taking the average for each group. Carefully choose explanatory variables by both biologically thinking and statistically examining: identify and exclude the variables that have high correlations with others. (3) Perform CCA using “Dataset to be explained” and explanatory variables. CCA regresses the former on the latter and thereby identifies the interpretable part of “Dataset to be explained” (designated as “Constrained data”). Subsequently CCA perfomes singular value decomposition of Constrained data and obtain “uncorrelated” axes (components). The axes are ordered by variation (eigenvalue), and first axes have largest eigenvalues. (4) Visualise the result of CCA using the first axes, and perform map analysis. Use two-dimensional map analysis before using three-dimensional plot (c.f. Fig. 1). First, interpret axes using a deep knowledge on the biological system and the experimental settings, although axes are not always interpretable [9]. It is generally helpful to analyse the relationships between axes and explanatory variables. Second, identify key sample clusters. Note that correlated elements gather towards the same direction from the origin. Strictly speaking, the size of the sample space is different from that of the gene space [9], therefore, do not use the Euclidean distance to measure the similarities between elements across different levels (e.g. cell samples and genes), but use angular distance instead to analyse the relationships between them. Third, explore key genes that are correlated with key clusters of samples and explanatory variables. (5) Lastly, design the next analysis. Optimise the combination of explanatory variables by performing CCAM using the same genes, and comparing %Explained of the analyses with different combinations of variables. It is also important to consider to remove unnecessary samples and/or genes, which can blunt the important difference between elements. The outputs of CCAM can be used as a scoring system.

### The analysis of variations in CCAM: decomposition of inertia

Fig. 6b summarises how the variation in “Dataset to be explained” is decomposed and retained in the result of CCAM. In the analysis using CA and CCA, the variation in data is measured by *inertia*, which plays the same role as the total variance in PCA. Technically, inertia is the sum of total Pearson 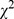 divided by the total sum [9]. Total inertia, 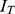, is decomposed into two parts, constrained inertia, 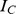, and unconstrained inertia (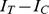). %Explained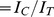 defines how much of the information in the original data is retained at this stage, and is useful for addressing the relevance of the findings by CCAM in terms of variation. Next, CCAM performs singular value decomposition, and constrained inertia is decomposed and distributed into new axes, 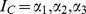.... %Visualised is defined as a ratio of (The sum of eigenvalues in the visualised space)
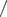
(Constrained inertia), and is useful to determine how many dimensions should be used. %Explained is comparable between the results from different combinations of explanatory variables, when the same genes are used and the main data matrix is the same.

#### Algebraic definition of CCAM

Suppose that the normalised gene expression of reference cell subsets (*environmental variables*) is 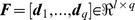, and the microarray data 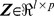. We assume that 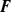 is standardized using 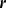 as weights for rows in the calculation of means and variances (see the constrained conditions of 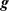 in the previous subsection). CA standardizes 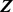 in the 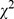-metric, and subsequently performs singular value decomposition (SVD) as in the following steps (Fig. S7): (1) Using 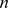, 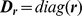 and 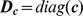 (see above), 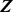 can be standardized in the 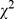-metric, 
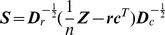
. (2) CCA projects 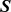 onto 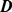, while probes are always weighted by the sums of rows of 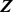. Thus, the projection matrix is 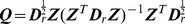, and the projected (constrained) matrix is 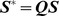. (3) Calculate the SVD of 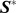: 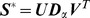 where 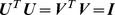, and 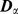 is the diagonal matrix of singular values in descending order (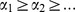). (4) Gene scores are given by 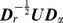, and sample scores are by 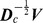. (5) Constrained inertias are 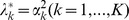, where 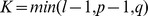. The percentage of the explained information in the 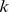-th axis is expressed by 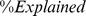 (of constrained inertia)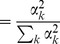. (6) In order to display environmental variables in triplots, environmental variables 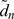 in 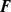 are linearly regressed to each axis of 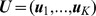. Suppose that 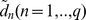 is standardized, the standardized regression coefficient 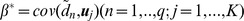, which is used for displaying environmental variables in triplots. (7) Using 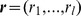 and 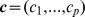, total inertia of the original gene expression matrix is 
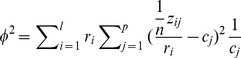
. 
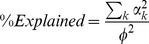
 is an estimate of how much of the original information is retained in the solution.

### Microarray data and processing

We have used GSE24759 for haematopoietic cell differentiation [30], GSE2779 and GSE15061 for MDS data (Table 1) [22], [49], GSE13159 for leukaemia data [29]. Microarray data were normalised by *rma* of the Bioconductor package, *affy*. Cross-platform comparisons were made by a commonly employed algorithm [60]. Batch-effects within each dataset, if any, were adjusted by an established approach using empirical Bayes methods [61]. Data were further normalised across genes. PCA was done by the function *dudi.pca* of the CRAN package *ade4*.

We used the CRAN package, *vegan*, for the computation of CCA [62]. An R script for CCAM is available as File S1. Microarray data of diseases and those of haematopoietic cell populations (environmental variables) were seprately normalised, as CCA analyses the data that were projected onto environmental variables, and normalisation of data of diseases and environmental variables did not have impacts on the results of CCA (data not shown). For sample scores, we employed *wa* score, and biplot arrows are based on weighted correlation of sample scores and haematopoietic cell differentiation [62], [63]. Genes were filtered by the same method used for gene signature, using haematopoietic cell data only, unless indicated. Thus, these analysis results are unsupervised for disease samples and do not use clinical information and sample identities. Sample scores of CCA results were first analysed by Kruskal-Wallis test for comparisons of multiple groups. Mann-Whitney U-test was used for the comparison of two groups. P-value was adjusted by the Bonferroni correction. Three-dimensional plots in Fig. 1e and Fig. S6 were produced by the R packages, *rgl* and *lattice*, respectively.

Provisional gene signatures were identified by a moderated, empirical Bayes t-statistic implemented in the Bioconductor package *limma* [64]. The top ranked genes (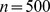) in comparison with all other cell populations by *topTable* of *limma* were designated as the provisional signature genes of each cellular population. Gene signature score was derived by analysing the averaged expression of signature genes and the gene expression of each disease sample by Pearson's correlation coefficients. Obtained scores were further scaled so as to have an average of zero and standard deviation of one, because the primary interest of the analysis is to compare between disease samples.

### Statistical analysis of survival data

Log-rank test was used for analysing the survival data of the stratified groups of patients. Cox proportional hazard models were used for analysis of survival data. The CRAN package, *survival*, was used for these computations.

## Supporting Information

Figure S1 Hierarchical clustering of gene expression signatures of haematopoietic cells by correlation to a leukaemia disease dataset. All the haematopoietic cell populations in GSE24759 were analysed. A gene signature was identified for each haematopoietic cell population, and gene signature score was calculated as a correlation between each signature and individual leukaemia sample, and shown by a heatmap. Yellow indicates positive correlation, and red indicates negative ones. Identified clusters of haematopoietic cell signatures are shown by colours of cell population names and dendrograms. Rows are individual patients. The disease categories are shown by colours on the left side of the heatmap. This analysis did not aim to classify disease samples, therefore reasonably, hierarchical clustering of rows did not classify disease samples. Abbreviations: CFU, colony forming unit; HSC, haematopoietic stem cell; CMP, common myeloid progenitor; GMP, granulocyte monocyte progenitor; MEP, megakaryocyte-erythroid progenitor.
(EPS)

Figure S2 Boxplots showing the distributions of gene signature score in leukaemic disease samples for indicated haematopoietic cell populations: (a) Mature B cell class switched; (b) Neutrophilic metamyelocyte; (c) Granulocyte-monocyte progenitor (GMP); and (d) Pro B cell. Positive values indicate that samples showed positive correlations with the corresponding gene signature.
(EPS)

Figure S3 CCAM results of AML patient samples using CMP, CFU-monocyte, Neutrophilic metamyelocyte, and mature Neutrophils as explanatory variables. (a) All the AML subtypes are displayed with haematopoietic cell data. (b) Individual disease subtypes are separately displayed with haematopoietic cell data.
(EPS)

Figure S4CCAM results of haematopoietic cell population data explained by major leukaemic phenotypes. CCAM was performed to analyse 38 hametopoietic cell populations using ‘average’ leukaemic cells (AML, ALL, CML, and CLL) as explanatory variables. (a) Disease and Cell plot. CCAM result of 38 haematopoietic cell populations (211 samples) is displayed with the average phenotypes of four major leukaemia subtypes. (b) Baricentres of haematopoietic cell populations are shown. Developmental pathways for erythrocyte, megakaryocyte, granulocyte, monocyte, and B cell lineages are shown by arrows. (c) Spider plots of selected cell lineages. The barycentre of each cell population is connected with individual cell samples, showing the distributions of each cell population. Dashed arrows show the developmental pathways of each cell lineage. It is not sound to use too many explanatory variables in CCAM, as CCAM regresses main data on explanatory variables, which is one of the major limitations of this method. Thus, in order to analyse the differentiation pathways of many normal haematopoietic cell populations in terms of the features of leukaemias, haematopoietic cell data has to be treated as main data, using leukaemic data as explanatory variables (note that this is the opposite way of Fig. 1). Map analysis indicates that the largest difference was observed between myeloid and lymphoid lineages (axis 1, a), and the second largest difference was between ALL and CML (axis 2, a). CCAM shows that (1) acute leukaemias (AML and ALL) are more associated with immature haematopoietic stem cells; (2) AML is more associated with immature myeloid lineage cells including CMP than differentiated myeloid cells; (3) CML is correlated with relatively differentiated monocytic and granulocytic lineage cells (especially, neutrophilic metamyelocyte and CFU-monocyte); (4) CLL is correlated with naive and mature B cells [31], [34]. (b–c). The results were compatible with and confirm at the transcriptomic level the common understandings on leukaemias and haematopoietic cell differentiation. Abbreviations: HSC1, Haematopoietic stem cells (CD133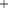 CD34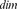); HSC2, Haematopoietic stem cells (CD38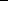 CD34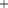); ERY1, Erythroid CD34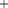 CD71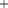 GlyA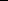; ERY2, Erythroid CD34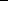 CD71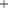 GlyA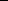; ERY3, Erythroid CD34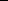 CD71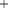 GlyA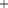; ERY4, Erythroid CD34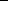 CD71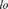 GlyA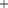; ERY5, Erythroid CD34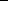 CD71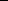 GlyA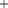; Mat-B1, Mature B cell class able to switch; Mat-B2, Mature B cell; Mat-B3, Mature B cell class switched; CD4 TCM, CD4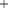 Central Memory T cell; CD4 TEM, CD4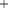 Effector Memory T cell; CD8 TCM, CD8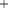 Central Memory T cell; CD8 TEM, CD8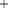 Effector Memory T cell; CD8 TEM RA, CD8
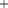
CD45RA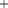 Effector Memory T cell; m-DC, Myeloid DC; p-DC, Plasmacytoid DC; NKa1, Mature NK CD56
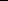
CD16
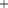
CD3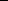; NKa2, Mature NK CD56
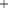
CD16
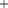
CD3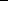; NKa3, Mature NK CD56
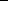
CD16
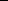
CD3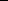.(EPS)

Figure S5 Comparison of the proposed method using CCA and a conventional multivariate analysis, principal component analysis (PCA). The CCA results are from the analysis in Fig. 3.
(EPS)

Figure S6 Three-dimensional plot of the HSC-CMP score, gene expression (average expression in each cell population), and four haematopoietic populations used for making the HSC-CMP score. CCAM assigned scores to both MDS disease samples and these genes. High scores were associated with MDS patients with poor prognosis (c.f. Fig. 4 and 5), while, at the gene level, genes with high scores showed higher expressions in HSC and MEP than GMP and CMP. On the other hand, genes with low scores are more highly expressed in CMP and GMP than HSC and MEP. See Table S1 for the relative contributions of those four haematopoietic populations to the HSC-CMP score.
(EPS)

Figure S7Graphical representation of the proposed method. Microarray data of undefined cells are standardized and designated as ***S***, and those of well-characterized cell subsets are preanalysed by PCA and Canonical Variate Criterion to produce 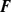. Generally, the number of environmental variable 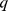 is much smaller than that of samples 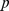 and genes 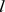, thus the number of new axes by singular value decomposition (SVD) is 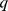.(EPS)

Table S1 Excel file of the list of genes that were used for constructing the HSC-CMP score, the gene scores of the CCAM result, and the biological/haematological features of these genes.
(XLS)

File S1 R script for CCAM.
(R) Click here for additional data file.

## References

[1] Liu R, Wang X, Chen GY, Dalerba P, Gurney A, et al (2007) The prognostic role of a gene signature from tumorigenic breast-cancer cells. N Engl J Med 356: 217–26. 17229949 10.1056/NEJMoa063994

[2] van 't Veer LJ, Dai H, van de Vijver MJ, He YD, Hart AA, et al (2002) Gene expression profiling predicts clinical outcome of breast cancer. Nature 415: 530–6. 11823860 10.1038/415530a

[3] Greenberg SA, Higgs BW, Morehouse C, Walsh RJ, Won Kong S, et al (2012) Relationship between disease activity and type 1 interferon- and other cytokine-inducible gene expression in blood in dermatomyositis and polymyositis. Genes Immun 13: 207–13. 21881594 10.1038/gene.2011.61

[4] Symmans WF, Hatzis C, Sotiriou C, Andre F, Peintinger F, et al (2010) Genomic index of sensitivity to endocrine therapy for breast cancer. J Clin Oncol 28: 4111–9. 20697068 10.1200/JCO.2010.28.4273 PMC2953969

[5] Tanaka RJ, Ono M, Harrington HA (2011) Skin barrier homeostasis in atopic dermatitis: feedback regulation of kallikrein activity. PLoS One 6: e19895. 21647431 10.1371/journal.pone.0019895 PMC3102059

[6] Sandberg R, Ernberg I (2005) Assessment of tumor characteristic gene expression in cell lines using a tissue similarity index (tsi). Proceedings of the National Academy of Sciences of the United States of America 102: 2052–2057. 15671165 10.1073/pnas.0408105102 PMC548538

[7] Koury M, Mahmud N, Rhodes M (2008) Wintrobe's Clinical Hematology, Lippincott Williams & Wilkins, chapter Origin and Development of Blood Cells. 12 edition.

[8] Larsson O, Wennmalm K, Sandberg R (2006) Comparative microarray analysis. OMICS 10: 381–97. 17069515 10.1089/omi.2006.10.381

[9] Greenacre M (2008) Correspondence Analysis in Practice. London: Chapman & Hall/CRC, 2nd edition.

[10] Tzeng J, Lu H, Li WH (2008) Multidimensional scaling for large genomic data sets. BMC Bioinformatics 9: 179. 18394154 10.1186/1471-2105-9-179 PMC2375126

[11] Wang HW, Trotter MWB, Lagos D, Bourboulia D, Henderson S, et al (2004) Kaposi sarcoma herpesvirus-induced cellular reprogramming contributes to the lymphatic endothelial gene expression in kaposi sarcoma. Nat Genet 36: 687–693. 15220918 10.1038/ng1384

[12] Iqbal J, Weisenburger DD, Greiner TC, Vose JM, McKeithan T, et al (2010) Molecular signatures to improve diagnosis in peripheral t-cell lymphoma and prognostication in angioimmunoblastic t-cell lymphoma. Blood 115: 1026–1036. 19965671 10.1182/blood-2009-06-227579 PMC2817630

[13] Kishino H, Waddell PJ (2000) Correspondence analysis of genes and tissue types and finding genetic links from microarray data. Genome Inform Ser Workshop Genome Inform 11: 83–95. 11700590

[14] Fellenberg K, Hauser NC, Brors B, Neutzner A, Hoheisel JD, et al (2001) Correspondence analysis applied to microarray data. Proc Natl Acad Sci U S A 98: 10781–6. 11535808 10.1073/pnas.181597298 PMC58552

[15] Baty F, Facompre M, Wiegand J, Schwager J, Brutsche MH (2006) Analysis with respect to instrumental variables for the exploration of microarray data structures. BMC Bioinformatics 7: 422. 17010189 10.1186/1471-2105-7-422 PMC1594581

[16] Bennett JM, Catovsky D, Daniel MT, Flandrin G, Galton DA, et al (1976) Proposals for the classification of the acute leukaemias. french-american-british (fab) co-operative group. Br J Haematol 33: 451–8. 188440 10.1111/j.1365-2141.1976.tb03563.x

[17] Griffin JD, Todd r R F, Ritz J, Nadler LM, Canellos GP, et al (1983) Differentiation patterns in the blastic phase of chronic myeloid leukemia. Blood 61: 85–91. 6571717

[18] Szczepanski T, van der Velden VH, van Dongen JJ (2003) Classification systems for acute and chronic leukaemias. Best Pract Res Clin Haematol 16: 561–82. 14592643 10.1016/s1521-6926(03)00086-0

[19] Sell S (2005) Leukemia: stem cells, maturation arrest, and differentiation therapy. Stem Cell Rev 1: 197–205. 17142856 10.1385/SCR:1:3:197

[20] Shih AH, Levine RL (2011) Molecular biology of myelodysplastic syndromes. Semin Oncol 38: 613–20. 21943667 10.1053/j.seminoncol.2011.04.013 PMC3183432

[21] Greenberg P, Cox C, LeBeau MM, Fenaux P, Morel P, et al (1997) International scoring system for evaluating prognosis in myelodysplastic syndromes. Blood 89: 2079–88. 9058730

[22] Mills KI, Kohlmann A, Williams PM, Wieczorek L, Liu WM, et al (2009) Microarray-based classifiers and prognosis models identify subgroups with distinct clinical outcomes and high risk of aml transformation of myelodysplastic syndrome. Blood 114: 1063–72. 19443663 10.1182/blood-2008-10-187203

[23] Vardiman JW, Thiele J, Arber DA, Brunning RD, Borowitz MJ, et al (2009) The 2008 revision of the world health organization (who) classification of myeloid neoplasms and acute leukemia: rationale and important changes. Blood 114: 937–51. 19357394 10.1182/blood-2009-03-209262

[24] Cazzola M (2011) Risk assessment in myelodysplastic syndromes and myelodysplastic/myeloproliferative neoplasms. Haematologica 96: 349–52. 21357714 10.3324/haematol.2010.030023 PMC3046263

[25] Malcovati L, Porta MG, Pascutto C, Invernizzi R, Boni M, et al (2005) Prognostic factors and life expectancy in myelodysplastic syndromes classified according to who criteria: a basis for clinical decision making. J Clin Oncol 23: 7594–603. 16186598 10.1200/JCO.2005.01.7038

[26] Wouters BJ, Lowenberg B, Delwel R (2009) A decade of genome-wide gene expression profiling in acute myeloid leukemia: ashback and prospects. Blood 113: 291–8. 18703705 10.1182/blood-2008-04-153239 PMC2615646

[27] Shaffer AL, Rosenwald A, Hurt EM, Giltnane JM, Lam LT, et al (2001) Signatures of the immune response. Immunity 15: 375–85. 11567628 10.1016/s1074-7613(01)00194-7

[28] Balgobind BV, Van den Heuvel-Eibrink MM, De Menezes RX, Reinhardt D, Hollink IH, et al (2011) Evaluation of gene expression signatures predictive of cytogenetic and molecular subtypes of pediatric acute myeloid leukemia. Haematologica 96: 221–30. 20971820 10.3324/haematol.2010.029660 PMC3031689

[29] Haferlach T, Kohlmann A, Wieczorek L, Basso G, Kronnie GT, et al (2010) Clinical utility of microarray-based gene expression profiling in the diagnosis and subclassification of leukemia: report from the international microarray innovations in leukemia study group. J Clin Oncol 28: 2529–37. 20406941 10.1200/JCO.2009.23.4732 PMC5569671

[30] Novershtern N, Subramanian A, Lawton LN, Mak RH, Haining WN, et al (2011) Densely interconnected transcriptional circuits control cell states in human hematopoiesis. Cell 144: 296–309. 21241896 10.1016/j.cell.2011.01.004 PMC3049864

[31] Chiorazzi N, Ferrarini M (2011) Cellular origin(s) of chronic lymphocytic leukemia: cautionary notes and additional considerations and possibilities. Blood 117: 1781–91. 21148333 10.1182/blood-2010-07-155663 PMC3056635

[32] Cox CV, Blair A (2005) A primitive cell origin for b-cell precursor all? Stem Cell Rev 1: 189–96. 17142855 10.1385/SCR:1:3:189

[33] Faderl S, Jeha S, Kantarjian HM (2003) The biology and therapy of adult acute lymphoblastic leukemia. Cancer 98: 1337–54. 14508819 10.1002/cncr.11664

[34] Lanasa MC, Weinberg JB (2011) Immunoglobulin class switch recombination in chronic lymphocytic leukemia. Leuk Lymphoma 52: 1398–400. 21699386 10.3109/10428194.2011.568076

[35] Burkle A, Niedermeier M, Schmitt-Graff A, Wierda WG, Keating MJ, et al (2007) Overexpression of the cxcr5 chemokine receptor, and its ligand, cxcl13 in b-cell chronic lymphocytic leukemia. Blood 110: 3316–25. 17652619 10.1182/blood-2007-05-089409

[36] Fournier S, Delespesse G, Rubio M, Biron G (1992) SarfatiM (1992) Cd23 antigen regulation and signalling in chronic lymphocytic leukemia. J Clin Invest 89: 1312–21. 1532590 10.1172/JCI115717 PMC442993

[37] Hubmann R, Duchler M, Schnabl S, Hilgarth M, Demirtas D, et al (2010) Notch2 links protein kinase c delta to the expression of cd23 in chronic lymphocytic leukaemia (cll) cells. Br J Haematol 148: 868–78. 19995395 10.1111/j.1365-2141.2009.08024.x

[38] Miura Y, Shimazu R, Miyake K, Akashi S, Ogata H, et al (1998) Rp105 is associated with md-1 and transmits an activation signal in human b cells. Blood 92: 2815–22. 9763566

[39] Porakishvili N, Memon A, Vispute K, Kulikova N, Clark EA, et al (2011) Cd180 functions in activation, survival and cycling of b chronic lymphocytic leukaemia cells. Br J Haematol 153: 486–98. 21443749 10.1111/j.1365-2141.2011.08605.x

[40] Anderson MK (2006) At the crossroads: diverse roles of early thymocyte transcriptional regulators. Immunol Rev 209: 191–211. 16448544 10.1111/j.0105-2896.2006.00352.x

[41] Blobel GA (2000) Creb-binding protein and p300: molecular integrators of hematopoietic transcription. Blood 95: 745–55. 10648382

[42] Hart SM, Foroni L (2002) Core binding factor genes and human leukemia. Haematologica 87: 1307–23. 12495904

[43] Martens JH (2011) Acute myeloid leukemia: a central role for the ets factor erg. Int J Biochem Cell Biol 43: 1413–6. 21664289 10.1016/j.biocel.2011.05.014

[44] Schoch C, Schnittger S, Klaus M, Kern W, Hiddemann W, et al (2003) Aml with 11q23/mll abnormalities as defined by the who classification: incidence, partner chromosomes, fab subtype, age distribution, and prognostic impact in an unselected series of 1897 cytogenetically analyzed aml cases. Blood 102: 2395–2402. 12805060 10.1182/blood-2003-02-0434

[45] Le Beau MM, Larson RA, Bitter MA, Vardiman JW, Golomb HM, et al (1983) Association of an inversion of chromosome 16 with abnormal marrow eosinophils in acute myelomonocytic leukemia. a unique cytogenetic-clinicopathological association. N Engl J Med 309: 630–636. 6577285 10.1056/NEJM198309153091103

[46] Schoch C, Haase D, Haferlach T, Freund M, Link H, et al (1996) Incidence and implication of additional chromosome aberrations in acute promyelocytic leukaemia with translocation t(15;17)(q22;q21): a report on 50 patients. British Journal of Haematology 94: 493–500. 8790148 10.1046/j.1365-2141.1996.d01-1829.x

[47] de Thé H, Chomienne C, Lanotte M, Degos L, Dejean A (1990) The t(15;17) translocation of acute promyelocytic leukaemia fuses the retinoic acid receptor alpha gene to a novel transcribed locus. Nature 347: 558–561. 2170850 10.1038/347558a0

[48] Nucifora G, Larson RA, Rowley JD (1993) Persistence of the 8;21 translocation in patients with acute myeloid leukemia type m2 in long-term remission. Blood 82: 712–715. 8338940

[49] Sternberg A, Killick S, Littlewood T, Hatton C, Peniket A, et al (2005) Evidence for reduced b-cell progenitors in early (low-risk) myelodysplastic syndrome. Blood 106: 2982–91. 16076868 10.1182/blood-2005-04-1543

[50] Nolte F, Hofmann WK (2010) Molecular mechanisms involved in the progression of myelodysplastic syndrome. Future Oncol 6: 445–55. 20222800 10.2217/fon.09.175

[51] Valent P, Wieser R (2009) Update on genetic and molecular markers associated with myelodysplastic syndromes. Leuk Lymphoma 50: 341–8. 19263296 10.1080/10428190902756107

[52] Nikpour M, Pellagatti A, Liu A, Karimi M, Malcovati L, et al (2010) Gene expression profiling of erythroblasts from refractory anaemia with ring sideroblasts (rars) and effects of g-csf. Br J Haematol 149: 844–54. 20408843 10.1111/j.1365-2141.2010.08174.x

[53] Ebert BL, Galili N, Tamayo P, Bosco J, Mak R, et al (2008) An erythroid differentiation signature 1 predicts response to lenalidomide in myelodysplastic syndrome. PLoS Med 5: e35. 18271621 10.1371/journal.pmed.0050035 PMC2235894

[54] Visvader JE (2011) Cells of origin in cancer. Nature 469: 314–22. 21248838 10.1038/nature09781

[55] Lane SW, Scadden DT, Gilliland DG (2009) The leukemic stem cell niche: current concepts and therapeutic opportunities. Blood 114: 1150–7. 19401558 10.1182/blood-2009-01-202606 PMC2723012

[56] Palmer MW (1993) Putting things in even better order: The advantages of canonical correspondence analysis. Ecology 74: 2215–2230.

[57] Ramette A (2007) Multivariate analyses in microbial ecology. FEMS Microbiology Ecology 62: 142–160. 17892477 10.1111/j.1574-6941.2007.00375.x PMC2121141

[58] Sugiman T (1998) Group dynamics in japan. Asian Journal of Social Psychology 1: 51–74.

[59] ter Braak C (1986) Canonical correspondence analysis: a new eigenvector technique for multivariate direct gradient analysis. Ecology 67: 1167–1179.

[60] Kuhn A, Luthi-Carter R, Delorenzi M (2008) Cross-species and cross-platform gene expression studies with the bioconductor-compliant r package ‘annotationtools’. BMC Bioinformatics 9: 26. 18201381 10.1186/1471-2105-9-26 PMC2267709

[61] Johnson WE, Li C, Rabinovic A (2007) Adjusting batch effects in microarray expression data using empirical bayes methods. Biostatistics 8: 118–27. 16632515 10.1093/biostatistics/kxj037

[62] Oksanen J, Blanchet FG, Kindt R, Legendre P, O'Hara RG, et al.. (2011) vegan: Community ecology package. Technical report. R package version 2.0–2.

[63] McCune B (1997) Inuence of noisy environmental data on canonical correspondence analysis. Ecology 78: 2617–2623.

[64] Smyth GK (2004) Linear models and empirical bayes methods for assessing differential expression in microarray experiments. Stat Appl Genet Mol Biol 3. 10.2202/1544-6115.1027 16646809

